# Rheumatoid arthritis serotype and synthetic disease-modifying anti-rheumatic drugs in patients with periodontitis: A case-control study

**DOI:** 10.1371/journal.pone.0252859

**Published:** 2021-06-21

**Authors:** Nik-Madihah Nik-Azis, Nurulhuda Mohd, Fazalina Mohd Fadzilah, Nor Hazla Mohamed Haflah, Mohd Shahrir Mohamed Said, Badiah Baharin

**Affiliations:** 1 Faculty of Dentistry, Department of Restorative Dentistry, Unit of Periodontology, Universiti Kebangsaaan Malaysia, Kuala Lumpur, Malaysia; 2 Radiology Department, Sunway Medical Centre, Bandar Sunway, Selangor, Malaysia; 3 Faculty of Medicine, Hospital Universiti Kebangsaan Malaysia, Bandar Tun Razak, Cheras, Kuala Lumpur, Malaysia; Nippon Medical School, JAPAN

## Abstract

Patients with rheumatoid arthritis (RA) experience a higher prevalence of periodontitis. This study aimed to examine the variation of periodontitis experienced with different serotypes suffered by RA patients and to examine the relationship between the different medications taken for RA that may influence this relationship. Two hundred and sixty RA and control participants underwent standardized periodontal examinations. Medical, serological and radiological (Sharp/van der Heijde) records were assessed. Functional status was assessed using the administered Health Assessment Questionnaire. Moreover, disease parameters, including disease activity (DAS28-ESR) and anti-citrullinated protein antibodies (ACPA) and rheumatoid factor (RF) seropositivity were evaluated. Periodontitis was higher in RA (71.54%) compared with controls (54.62%). The stage of periodontitis experienced by ACPA-positive participants were higher than APCA-negative participants. The probing pocket depth and recession experienced by RF-positive participants were higher than those who were RF-negative. RA participants on methotrexate had lower clinical attachment loss and lower periodontal probing depth compared with participants on a combination methotrexate and other disease-modifying antirheumatic drugs. Participants taking corticosteroids had lower gingival index scores. The association between seropositivity and the type of medications taken with periodontal health parameters in this group of patients suggests that both seropositivity and medications taken are important modifiers in the relationship between periodontitis and RA.

## Introduction

Periodontitis is a disease that affects the supporting structures of the teeth. It is characterised by microbially-associated, host-mediated inflammation that results in the loss of periodontal attachment [1]. Rheumatoid arthritis (RA) is an autoimmune disease with chronic inflammation characterised by joint swelling, joint tenderness and destruction of synovial joints [2]. Both periodontitis and RA are multifactorial complex diseases with many similarities between them including their common mechanisms of chronic inflammation and bone destruction [3].

Auto-antibodies to the fragment crystallizable (Fc) portion of the immunoglobulin are known as rheumatoid factor (RF) while antibodies that form against citrullinated proteins are called anti-citrullinated protein antibodies (ACPA) [4]. The relationship between RA and periodontitis was hypothesised to be due to the role of the periodontal pathogen *Porphyromonas gingivalis* in the production of the peptidylarginine deiminase enzyme (PAD). This PAD enzyme could break immune tolerance and trigger a latent antibody response against citrullinated proteins prior to the onset of RA. *Porphyromonas gingivalis* is the only known microorganism to produce the enzyme PAD although the human PAD, and the bacterial PAD differ in terms of their enzymic activity [5]. This biological mechanism would result in a higher prevalence and severity of periodontitis in ACPA-positive RA participants. RF-positive RA patients have also been found to be more likely to have moderate to severe periodontitis than patients who were RF-negative [6]. However, there are currently limited information on the relationship of these auto-antibodies especially RF and periodontitis.

Periodontitis involves a dysregulation of both the inflammatory and the immune pathways. The conventional synthetic disease-modifying antirheumatic drugs (DMARDs) taken for RA are immunomodulatory and can have anti-inflammatory effects [7]. This may improve periodontal health parameters. However, there are very limited studies on DMARDs and periodontal health. One study reported beneficial effects of these medications when used as an adjunct following periodontal treatment [8] while another study found no such difference [9]. The conflicting findings may be due to the different effects of the individual DMARDs and combinations of DMARDs on the periodontium. This was demonstrated by Romero-Sanchez et al. [10] where participants on methotrexate combined with leflunomide were found to have a more severe form of periodontitis compared to participants taking other DMARDs, suggesting that different DMARDs may have different effects on the periodontium.

It was hypothesised that a) the prevalence and severity of periodontitis in RA participants will be higher than the control group; b) seropositive RA patients will have a greater prevalence and severity of periodontitis, supporting the biological hypothesis linking RA and periodontitis; and c) there is a difference in the prevalence and severity of periodontitis participants taking different types of conventional synthetic DMARDs. Hence, this study aimed to a) investigate the prevalence and severity of periodontitis in RA participants; b) examine the variation of periodontitis suffered by RA patients with different ACPA and RF serotypes and c) assess the associations between the different medications taken for RA with periodontal health parameters.

## Methods

This was a case-control study on periodontitis in RA participants with osteoarthritis (OA) participants as the control group. The use of OA participants was based on the rationale that they will have more similar sociodemographic characteristics and is from the same study base as the RA participants to ensure that the inferential validity of the study in not compromised [11,12]. Ethical approval for the study was obtained from Ethical Board of the Universiti Kebangsaan Malaysia (UKM/PPI/111/8/JEP-2017-553). Reporting of this study was made in accordance with the STROBE guidelines. The RA participants were recruited from the Rheumatoid Arthritis Clinic, whereas the OA participants were recruited from the Osteoarthritis Clinic both in Hospital Canselor Tuanku Mukhriz, Kuala Lumpur from October 2017 until October 2018. Recruitment was performed by a single researcher (NMNA) where consecutive patients meeting the inclusion and exclusion criteria was invited to join the study.

### Inclusion and exclusion criteria

The inclusion criteria were as follows: i) RA patients, as confirmed by the American College of Rheumatology (ACR) -European League Against Rheumatism (EULAR) Classification [2] or OA as confirmed by the ACR Classification [13–15]; (ii) above the age of 18 years old; iii) dentate; and iv) able to give verbal and written consent.

The exclusion criteria were as follows: (1) patients who were unable to read, write, or understand Malay or English; (2) coexistence of other autoimmune diseases; (3) uncontrolled systemic disease or malignancy; (4) patients who were pregnant or planning to become pregnant; (5) patients who were currently undergoing or had previous history of periodontal treatment, including root surface debridement and/or periodontal surgery; and (6) previous or current use of phenytoin or cyclosporin.

### Sample size calculation

The sample size calculation was conducted using the OpenEpi Version 3.01. The primary objective of investigating the prevalence of periodontitis in RA participants compared with controls was used for the sample size calculation. The null hypothesis was that the odds ratio is equal to 1. The probability (power) was selected to be 0.8. The ratio of sample size in exposed/unexposed group was 1. Prior data indicated that the probability of exposure in the population is 0.485 (NOHSA, 2010). True odds ratio for disease in exposed subjects relative to unexposed subjects was estimated at 2.1 [6]. The sample size recommended was 130 for both groups.

### Data collection

The demographic information was first obtained from the patient using measurements taken during recruitment. Three categories of data were then collected. The first category was the periodontal parameters obtained from a clinical examination of the participants. A Health Assessment Questionnaire (HAQ) was later administered to ascertain the functional status of the participants. Finally, medical notes and serological results were accessed to extract information regarding the disease severity, disease activity and treatment, which included prescribed medications. The extracted information was verified to ensure that they were dated no longer than three months in duration from the clinical examination.

### Oral examination

Prior to the initiation of the study, the examiner (NMNA) was calibrated against a gold-standard periodontist (NM). The assessment of the periodontal health used a combination of indices, such as the number of remaining teeth, the plaque index [16], the gingival index [17], the probing pocket depth (PPD) and clinical attachment loss (CAL). Diagnosis of periodontitis was made based on the criteria outlined by Papapanou et al. [18].

### RA parameters

For the RA participants, the parameters assessed were as follows: a) disease activity and severity using the Disease Activity Score 28-Erythrocyte Sedimentation Rate (DAS28-ESR); b) radiographic damage indices using the modified Sharp/van der Heijde method; [19] c) seropositivity based on the RF and/or ACPA serum titre results; and d) functional status, as assessed by the Malaysian HAQ [20].

### Statistical analysis

Statistical analysis of variables was performed with IBM SPSS version 19.0 (IBM Co., Armonk, NY, USA). Demographic data were presented as means ± standard deviations (± SD) for continuous measures and frequencies (%) for all discrete variables. Univariate comparisons were made using the Student t-test or Chi-square test as appropriate. The correlations between periodontal indices and RA disease activity/characteristics were analysed by Pearson or Spearman correlation coefficients, as appropriate. Mann-Whitney U test was applied for the analysis of independent nonparametric variables. A multiple logistic regression analysis was carried out to further investigate the relationships of an RA diagnosis with periodontitis stage and other variables. All *p* values were two-sided, and *p* values less than 0.05 were considered statistically significant.

## Results

### Sociodemographic data

**Fig 1:**
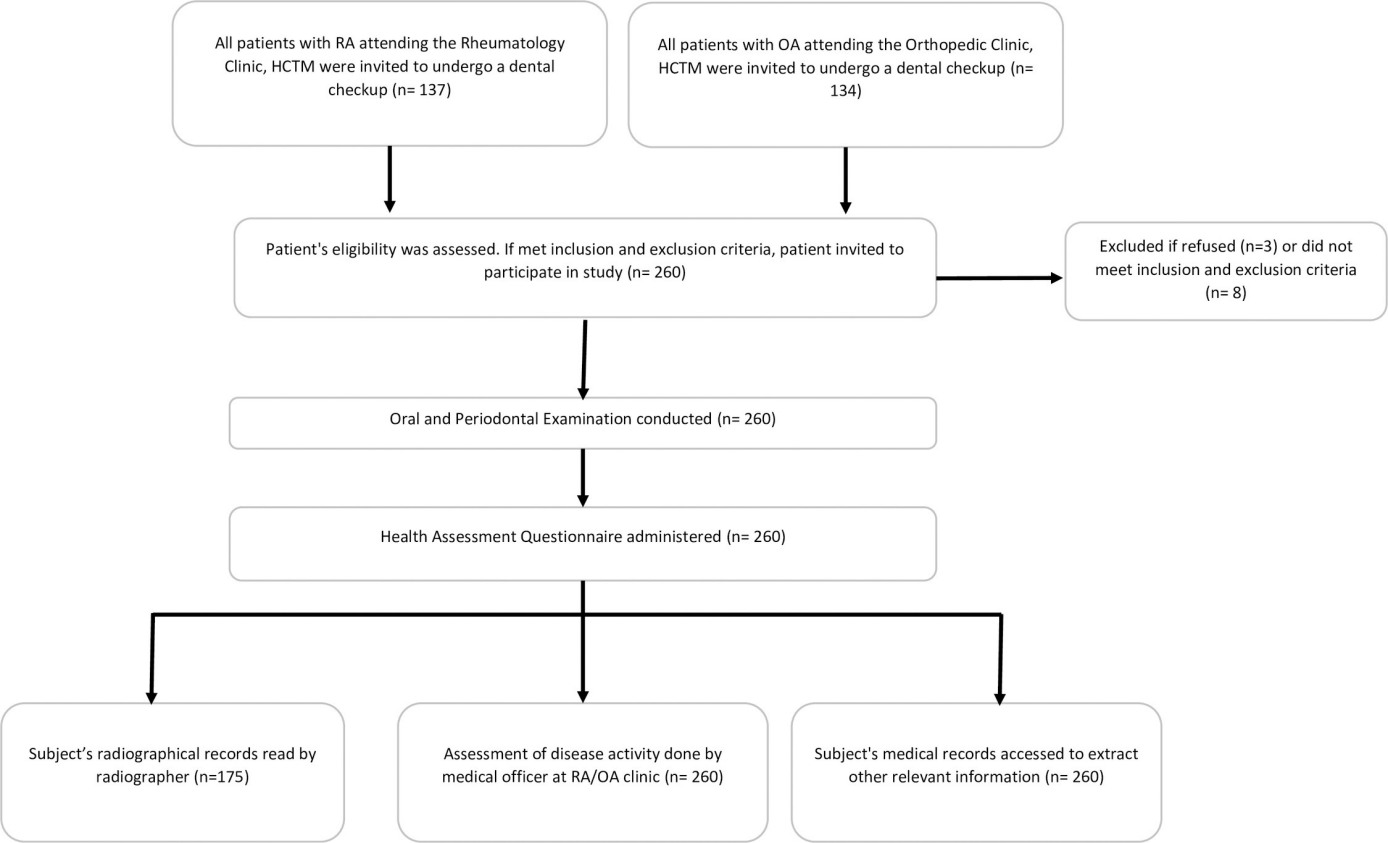
Flow diagram of the recruitment and data collection of the participants.

Out of the 260 participants recruited, 130 participants were from the RA Clinic, and 130 participants were from the OA Clinic. Fig 1 shows the recruitment process and the number of samples for each of the stages of the data collection. For the RA seropositivity, data on ACPA status were available for 46 participants and RF for 114 participants. All other parameters investigated were complete at 130 for each group.

The demographic characteristics for the participants are shown in Table 1. There was no difference between the demographic characteristics of the two groups, except for the proportion of participants whose highest level of education was primary education.

**Table 1:** Demographic data for study participants.

		RA (n = 130)	OA (n = 130)	*p*-value
Gender	Female	119	106	0.386
Race	Malay	66	74	0.499
	Chinese	43	40	0.742
	Indian	20	14	0.303
	Other	1	2	0.564
Age	Age	56.6	61.5	0.999
Education	Primary	28	48	0.022*
	Secondary	64	53	0.309
	Tertiary	38	29	0.272
BMI (mean ± SD)	Kg/m^2^	26.5 (± 6.1)	28.4 (± 5.1)	0.177
Smoking	No	122	121	0.949
	Past	5	4	0.739
	Current	3	5	0.480
Diabetes	Yes	30	44	0.104
	No	100	86	0.305
NSAIDS	Yes	52	53	0.899

### Prevalence and severity of periodontitis in RA and OA participants

Ninety-three (71.54%) RA participants and 71 (54.62%) controls had periodontitis. The Chi-squared test was statistically significant (χ2 (1, N = 260) = 7.28, *p* = 0.007) where the prevalence of periodontitis in RA participants was significantly higher than in controls. The chi-squared test was also significant for the stage of periodontitis, χ2 (3, N = 164) = 8.08, *p* = 0.044. There was no significant difference in the grades of periodontitis between the RA and OA subjects. The periodontal health parameters for the RA and control groups of participants are shown in Table 2. RA participants had greater average probing pocket depth and lower gingival index compared to the controls.

**Table 2:** Stage and grade of periodontitis and periodontal health parameters in cases and controls.

	RA patients		OA controls		*p*-value
	N	%	N	%	
**Stage of Periodontitis**					
Stage 1	30	23.1	17	13.1	0.044*
Stage 2	37	28.5	19	14.6	
Stage 3	17	13.1	21	16.2	
Stage 4	9	6.9	14	10.8	
**Grade of Periodontitis**					
Grade A	4	4.3	6	8.5	0.451
Grade B	82	88.2	58	81.7	
Grade C	7	7.5	7	9.9	
**Periodontal Health Parameters**					
	**N**	**Median (Q**_**1**_**-Q**_**3**_**)**	**N**	**Median (Q**_**1**_**-Q**_**3**_**)**	***p*-value**
Number of Teeth	130	22.0 (14.0–26.0)	130	21.5 (12.8–25.2)	0.377
DMFT	130	13.0 (3.0–18.0)	130	12.0 (7.0–18.0)	0.436
Plaque Index	130	36.5 ± (16.0–100.0)	130	40.0 (15.0–71.3)	0.830
Gingival Index	130	10.5 (5.0–100.0)	130	37.5 (10.0–60.0)	0.000*
Average PPD	130	2.1 (1.8–2.9)	130	1.7 (1.0–2.9)	0.000*
Average CAL	130	3.0 (2.0–4.3)	130	3.0 (1.5–4.4)	0.383

DMFT- Decayed, missing, filled teeth; PPD- Probing pocket depth; CAL- Clinical attachment loss.

To investigate the relationships of an RA diagnosis with periodontitis stage and other variables, multiple logistic regression was used with the following characteristics examined as predictors: periodontitis stage (none, stages 1 and 2 and stages 3 and 4), smoking (yes/no), HbA1c, age, race, gender, BMI, education level (primary, secondary and tertiary) and plaque index. Race, smoking status and plaque index were not significantly associated with RA diagnosis in the univariable analysis and were not included in the final model.

The omnibus model for the logistic regression analysis was statistically significant, χ2 (6, N = 260) = 50.758, *p* = 0.000 (*p* < 0.05), Cox & Snell R2 = 0.233, Nagelkerke R2 = 0.317. The model was 72.3% accurate in its predictions of RA diagnosis compared with OA. The odds ratio of having RA compared with controls was 2.80 times higher (95% confidence interval [CI] = 1.25 to 6.26) for each one-step increase in periodontitis stage (from none to Stage 1 and 2 to Stage 3 and 4) while controlling for gender, age, HbA1c and BMI.

### RA disease activity, radiographic findings and functional limitations

For the DAS-28 scores, the mean score was 3.32 ± 0.817 (N = 130). As for the Sharp/Van der Heijde score, the mean was 19.07 ± 20.39 (N = 54). Table 3 shows the correlation between the RA parameters compared to the periodontitis, periodontitis stages, and periodontal health parameters. There was correlation between the RA disease duration, DAS-28 score, HAQ and Sharp/Van der Heijde score with the teeth count. There was no correlation between the RA disease activity as measured by DAS28-ESR and periodontitis severity as measured by the stage of periodontitis and the average PPD.

**Table 3:** Correlation between the RA parameters compared to the PD parameters.

	PD Diagnosis	Periodontitis Stage	Teeth Count	Plaque Index	Gingival Index	Average PPD
**RA Disease duration; r**_**s**_ **(*p*-value)**	0.131 (0.035*)	-0.163 (0.038*)	-0.202 (0.001*)	0.064 (0.306)	-0.086 (0.169)	0.121 (0.051)
**DAS28-ESR; r**_**s**_ **(*p*-value)**	-0.179 (0.042*)	0.015 (0.886)	-0.222 (0.011*)	0.036 (0.682)	-0.040 (0.653)	-0.076 (0.391)
**HAQ; r**_**s**_ **(*p* value)**	-0.075 (0.230)	0.157 (0.044*)	-0.174 (0.005*)	0.080 (0.198)	0.007 (0.913)	0.027 (0.661)
**Sharp/van der Heijde; r**_**s**_ **(*p*-value)**	-0.030 (0.828)	-0.029 (0.833)	-0.399 (0.003*)	0.258 (0.059)	-0.074 (0.596)	0.076 (0.584)

PD Diagnosis was categorised to whether subjects had healthy gingiva, gingivitis or periodontitis; the RA parameters were continuous variables using the overall score of the parameter.

### Periodontal health parameters according to the rheumatoid arthritis serotype

The amount and percentages of the participants that suffered from periodontitis and the breakdown of their stage and grade of periodontitis according to their serotype are shown in Table 4. The Chi-squared test showed a statistically significant difference between the stage of periodontitis experienced by ACPA-positive and ACPA-negative participants (χ2 (3, N = 46) = 10.28, *p* = 0.016). No difference in the stage and grade of periodontitis was found between participants who were RF-positive and RF-negative.

**Table 4:** Diagnosis, stage and grade of periodontitis according to ACPA and RF status for RA subjects.

	Subjects with ACPA Data (n = 46)			Subjects with RF Data (n = 114		
	ACPA +ve (n = 31)	ACPA -ve (n = 15)	*p-*value	RF +ve (n = 79)	RF -ve (n = 35)	*p-*value
Periodontitis; n (%)	21 (67.7)	11 (73.3)	0.699	55 (69.6)	25 (71.4)	0.846
Stage 1; n (%)	6 (19.4)	4 (26.7)	0.016*	17 (30.9)	9 (36)	0.076
Stage 2; n (%)	3 (9.7)	6 (40.0)		18 (32.7)	13 (52)	
Stage 3; n (%)	11 (35.5)	0 (0)		16 (29.1)	1 (4.0)	
Stage 4; n (%)	1 (3.2)	1 (6.7)		4 (7.3)	2 (8.0)	
Grade A; n (%)	2 (9.5)	1 (9.1)	0.891	1 (1.8)	2 (8.0)	0.218
Grade B; n (%)	18 (85.7)	9 (81.8)		51 (92.7)	20 (80.0)	
Grade C; n (%)	1 (4.8)	1 (9.1)		3 (5.5)	3 (12.0)	

Participants who were RF-positive had greater probing pocket depth (U = 1033.5, z = -2.14, *p* = 0.032) and recession depth (U = 1045.5, z = -2.24, *p* = 0.025) compared to those who are RF-negative. There was no other difference in the periodontal health parameters according to RA seropositivity.

### Disease-modifying antirheumatic drugs and other medications

Out of all the RA participants, 109 participants were taking DMARDs. All were taking conventional synthetic DMARDs, with no subjects on biological DMARDs. Most subjects on monotherapy were taking methotrexate (28.5%). There was no difference in any of the periodontal health parameters and the RA parameters between the participants taking different types of medications. This is shown in Table 5.

**Table 5:** Periodontal health parameters and RA parameters according to the DMARDs subgroups.

Medication Type	MTX	HCQ	SSZ	LFM	Comb	No	*p*-value
N (%)	37 (28.5)	4 (3.1)	8 (6.2)	3 (2.3)	57 (43.8)	20 (15.4)	NR
Periodontal Health Parameters							
Periodontal Diagnosis							0.354
Healthy; n (%)	9 (7.0)	2 (1.6)	2 (1.6)	1 (0.8)	12 (9.3)	5 (3.9)	
Gingivitis; n (%)	1 (0.8)	0	2 (1.6)	0	3 (2.3)	0	
Periodontitis; n (%)	27 (20.9)	2 (1.6)	4 (3.1)	2 (1.6)	42 (32.6)	15 (11.6)	
Periodontitis Stage							0.107
Stage 1; n (%)	10 (10.9)	0	0	1 (1.1)	11 (12.0)	7 (7.6)	
Stage 2; n (%)	15 (16.3)	1 (1.1)	3 (3.3)	1 (1.1)	12 (13.0)	5 (5.4)	
Stage 3; n (%)	1 (1.1)	0	1 (1.1)	0	13 (14.1)	2 (2.2)	
Stage 4; n (%)	1 (1.1)	1 (1.1)	0	0	6 (6.5)	1 (1.1)	
Teeth Count; (mean ± SD)	20.54 ±5.99	6.50 ±4.93	18.88 ±10.62	20.67 ±6.03	19.74 ±7.96	20.95 ±6.48	0.532
Plaque Index; (mean ± SD)	36.28 ±37.64	65.75 ±33.93	63.25 ±34.73	29.00 ±26.21	54.83 ±36.28	40.75 ±29.29	0.243
Gingival Index; (mean ± SD)	26.89 ±37.58	37.25 ±30.36	38.88 ±32.24	4.67 ±3.51	27.86 ±36.60	19.10 ±28.97	0.265
Average PPD; (mean ± SD)	2.18 ±0.89	2.21 ±1.34	2.43 ±1.21	2.07 ±0.07	2.54 ±0.96	2.21 ±0.73	0.208
Average CAL; (mean ± SD)	2.75 ±1.48	2.21 ±1.34	3.81 ±3.03	2.07 ±0.07	3.91 ±1.94	3.11 ±1.34	0.052
RA Parameters							
RA Disease duration; (mean ± SD)	12.51 ±11.02	13.00 ±7.70	16.63 ±18.35	13.67 ±9.29	10.54 ±8.58	11.15 ±9.62	0.930
DAS28-ESR; (mean ± SD)	3.22 ±0.81	3.38 ±1.27	3.06 ±0.88	3.81 ±1.56	3.36 ±0.80	3.39 ±0.73	0.690
HAQ; (mean ± SD)	0.25 ±0.46	1.03 ±1.34	0.56 ±0.83	1.46 ±1.26	0.46 ±0.70	0.44 ±0.73	0.246
Sharp/van der Heijde; (mean ± SD)	15.27 ±21.90	65.00	2.67 ±6.53	14.00	22.38 ±18.51	22.70 ±22.59	0.055

SD- standard deviation; MTX- methotrexate; HCQ- hydroxychloroquine; SSZ- sulfasalazine; LFM- leflunomide; Comb- combination of more than one DMARDS; No- not on any DMARDs; NR-not relevant.

To further investigate the difference between participants on methotrexate, comparison between the periodontal parameters and RA parameters of subjects on methotrexate alone (MTX-mono) compared to those on methotrexate in combination with other DMARDs (MTX-combo) was carried out as shown in Table 6. The chi-squared test was statistically significant for the stage of periodontitis, χ2 (3, N = 64) = 14.793, *p* = 0.002 where subjects on a combination of methotrexate and other DMARDs had higher stage of periodontitis compared to those on monotherapy with methotrexate. Subjects on a combination of methotrexate and other DMARDs also showed significantly worst periodontal health parameters with higher plaque index (U = 628.00, z = -2.397, *p* = 0.017), deeper PPD (U = 589.50, z = -2.712, *p* = 0.007) and deeper CAL (U = 448.00, z = -3.960, *p* = 0.000).

**Table 6:** Periodontal health parameters and RA parameters according to the methotrexate monotherapy or methotrexate in combination with other DMARDs.

Medication Type	MTX-mono	MTX-combo	*p*-value
N (%)	39 (30.0)	46 (35.4)	NR
Periodontal Health Parameters			
Periodontal Diagnosis			0.851
Healthy; n (%)	9 (23.1)	9 (19.6)	
Gingivitis; n (%)	1 (2.6)	2 (4.3)	
Periodontitis; n (%)	29 (74.4)	35 (76.1)	
Periodontitis Stage			0.002*
Stage 1; n (%)	11 (37.9)	7 (20)	
Stage 2; n (%)	16 (55.2)	10 (28.6)	
Stage 3; n (%)	1 (3.4)	13 (37.1)	
Stage 4; n (%)	1 (3.4)	5 (14.3)	
Teeth Count; (mean ± SD)	20.74 ± 5.92	20.72 ±7.46	0.577
Plaque Index; (mean ± SD)	36.37 ±36.76	53.45 ±37.05	0.017*
Gingival Index; (mean ± SD)	25.95 ±36.38	28.57 ± 36.18	0.746
Average PPD; (mean ± SD)	2.15 ±0.88	2.63 ±0.96	0.007*
Average CAL; (mean ± SD)	2.69 ±1.46	4.15 ±1.97	0.000*
RA Parameters			
RA Disease duration; (mean ± SD)	12.59 ±10.98	9.07 ±7.74	0.138
DAS28-ESR; (mean ± SD)	3.23 ±0.83	3.35 ±0.81	0.385
HAQ; (mean ± SD)	0.29 ±0.53	0.40 ±0.67	0.535
Sharp/van der Heijde; (mean ± SD)	14.85 ±20.10	20.75 ±19.02	0.289

MTX-mono- Methotrexate alone; MTX-combo- Methotrexate in combination with other DMARDs; PPD- periodontal probing depth; CAL- clinical attachment loss; HAQ- health assessment questionnaire; SD- standard deviation.

Other than the DMARDs, participants were also taking NSAIDs (40.0%) corticosteroids (45.4%) and bisphosphonates (3.0%). The associations between participants taking NSAIDs, corticosteroid and bisphosphonates medications and their oral health parameters were investigated using the Mann Whitney test. The Mann-Whitney U test indicated that the gingival index was significantly lower (U = 4909.5, z = -2.02, *p* = 0.04) in subjects taking corticosteroids (mean rank = 113.21, n = 260) compared to subjects not on corticosteroids (mean rank = 135.57, n = 260). No other difference was found in the other periodontal health parameters.

## Discussion

This was a study comparing periodontitis in RA participants to OA controls. To date, only limited studies using a similar control group [6,21–23]. The National Oral Health Survey 2010 for Malaysia estimated that 48.5% of adults in Malaysia have periodontitis [24]. Hence, the prevalence of participants with periodontitis in this study was generally higher than the national average where 71.5% of RA participants and 51.6% of controls had periodontitis. The poorer periodontal health of the RA participants in this study can be attributed to many factors, including the poor general health of the participants. RA weakens the immune defence in the host and causes an increase in the host’s systemic inflammation. They can also have functional and nutritional limitations [25]. This can enhance the severity of periodontitis experienced by RA participants [26]. The presence of RA also increases the risk of infections, and this risk is increased further with the use of DMARDs [27].

This study found the stage of periodontitis experienced by ACPA-positive participants to be more severe compared with ACPA-negative participants. Other studies with similar outcomes, including those from the United States of America [21], Korea [28], Japan [29], India [30] and Spain [31] found that the presence of periodontitis was associated with greater RA disease activity. The odds ratio of RA patients with periodontitis presenting with an increased RA disease activity has been reported to be 2.9 [32]. Associations between PD parameters and positivity and levels of ACPA was found not only in RA participants, but also in a healthy population as reported by the Japanese study [29]. In contrast, a Swedish study found that the prevalence of periodontal treatment codes did not differ between ACPA-positive and ACPA-negative RA [33]. Other more recent studies reporting no differences in ACPA levels among RA patients according to their periodontal status includes those from Sweden [34,35], Indonesia [36], Spain [22] and Bulgaria [37].

The findings of participants with a seropositive ACPA having a more severe periodontitis stage supports the biological mechanism linking these two diseases. The presence of *Porphyromonas gingivalis* in the periodontitis participants produces the *Porphyromonas gingivalis* PAD enzymes. The PAD could be a factor that breaks the subject’s immune tolerance and trigger a latent antibody response against citrullinated proteins prior to the onset of RA.

Although studies as early as in the 1980s have reported RF findings in subgingival plaque, inflamed gingiva, stimulated pooled saliva and serum of patients with chronic moderate periodontitis, there are very limited studies on RF and periodontitis [38]. Chronic inflammation in periodontitis appears to significantly increase the formation of RF; with the RF in periodontitis patients showing a cross-reaction with oral bacterial epitopes [39,40]. This study found that RF-positive participants had greater probing pocket depth and recession. This is similar to another study where patients with RA who were RF-positive were found to be more likely to have moderate to severe periodontitis than patients who were RF-negative [6]. Unlike ACPA seropositivity and periodontitis, there is currently no proposed mechanism linking RF and periodontitis.

A relationship between RA clinical disease activity and PD severity has been reported in other studies. Mikuls et al [21] found the presence of PD was associated with increase increased swollen joint counts, greater RA disease activity, higher radiographic damage of joints and increased ACPA and RF levels. This finding is supported by Rodríguez-Lozano et al [22] where periodontitis severity was significantly associated with RA disease activity. This study however found no association between RA clinical disease activity and PD severity.

This study is one of very few studies reporting on the different synthetic DMARDs taken by RA participants and its correlations with periodontal health parameters. In this study, participants taking a combination of methotrexate and other DMARDs had significantly higher CAL, PPD and plaque index compared with those taking methotrexate alone. This is similar to reports where participants on methotrexate combined with leflunomide exhibited a higher extension of CAL in their study [10]. The findings can be due to the different mechanisms of action of the different DMARDs, each with a varying effect on the periodontal tissues. This can also be explained by the pattern of prescription where methotrexate is prescribed to participants newly diagnosed with RA [41,42]. Participants with more advanced RA have usually first tried monotherapy unsuccessfully and will be on a combination of medications [43]. Hence, a higher PPD and CAL in participants on a combination of DMARDs suggests that participants with a more severe form of RA has a more severe form of periodontitis. Interaction of inflammatory mediators from periodontal inflammation can also potentially affect the efficacy of the DMARDs prescribed, further contributing to the type and amount of DMARDs taken by RA subjects with PD [44].

Participants on systemic corticosteroids was found to exhibit a significantly lower gingival index compared with participants not taking any corticosteroids. The anti-inflammatory and immune suppression properties of corticosteroids could explain this outcome. This is in line with another report that steroid therapy altered the gingival bleeding index in a dose-related manner [45].

Data on the ACPA status for 46 participants and RF status for 114 participants was available in this study. This is because the ACPA test was not available on-site and had to be performed outside of the hospital. While data on the type of medications for RA taken by the participants were collected, the information on other medications prescribed were not studied including the adherence to the medications. This too can influence the clinical presentation of both the RA and the periodontitis [46]. The restricted information on the ACPA status and the medications prescribe is a limitation of this study.

This study that adopted the most updated definition based on the 2018 case definition by AAP/EFP for periodontitis [18], whereas other studies used varying definitions including the case definitions by Eke et al. [47]. This may lead to inaccuracies when results are directly compared especially since there are no comparable reports yet on the diagnosis, stage and grade of periodontitis in RA patients based on the new classifications.

## Conclusion

A high proportion of RA patients are afflicted with periodontitis compared to the controls. ACPA and RF serotype and the medications taken by the RA patients are associated with some periodontal health parameters and may impact on the periodontal health of this group of patients. It is suggested that clinicians treating RA patients with periodontitis to consider these two factors when diagnosing and formulating a treatment plan for the management of periodontitis in this group of patients.

## Supporting information

S1 File Anonymous data sets collected and analysed for this study. (SAV)
